# On the quality of reporting quality of life after extracorporeal life support

**DOI:** 10.1177/02676591221127562

**Published:** 2022-09-15

**Authors:** Lara C A Pladet*, Christiaan L Meuwese, Olaf L Cremer, Dirk W Donker

**Affiliations:** 1 Department of Intensive Care Medicine, 8124 University Medical Center Utrecht, Utrecht, The Netherlands; 2 Department of Intensive Care Medicine, 6993 Erasmus Medical Center, Rotterdam, The Netherlands; 3 Department of Cardiology, 6993 Erasmus Medical Center, Rotterdam, The Netherlands; 4 TechMed Centre, Cardiovascular and Respiratory Physiology, University of Twente, Enschede, The Netherlands

Dear editor:

With great interest we read the recent study by de Vlugt et al. investigating the effect of extracorporeal life support (ECLS) on 3-months quality of life and neuropsychological symptoms. There were no major differences observed between ECLS supported patients versus matched non-ECLS patients admitted to the intensive care unit (ICU).^1^ These findings suggest that the long-term sequelae of ECLS treatment are limited. Despite the importance of the topic,^2^ we feel that there are some relevant methodological issues that should be considered when interpreting the results.

The small number of patients (19 ECLS vs 38 non-ECLS) limit the reliability of results, as it renders the study underpowered to detect clinically meaningful differences in patient-centered outcomes. In addition, as the analyzed cases represented a highly-particulate subgroup of 19 (27%) patients for whom outcome data were available (selected from a total of 70 ECLS survivors), a high concern for observation bias arises. For example, individuals in a poor health state are well known to participate less frequently in surveys.^3^ Such effects could have led to an underestimation of poor quality of life and neuropsychological outcomes in this study. In addition, several patient characteristics including female gender, higher socioeconomic status and increasing age were described to increase response rates of EQ5D questionnaires in literature.^4^ To objectively evaluate the potential for bias, it is important to compare the response rates of ECLS patients to those of the matched ICU patients. However, we were not able to find information about completeness of questionnaires in the control group. Also, potential differences between responders and non-responders were not reported.

Another point of concern is related to the high likelihood for residual confounding. Matching only occurred on diagnosis group. Of note, Sequential Organ Failure Assessment (SOFA) and Acute Physiology And Chronic Health Evaluation (APACHE) IV scores both do not take into account the time-dependent confounding that occurs due to evolution of disease severity later during the ICU stay. Indeed, important differences between ECLS patients and the matched ICU group were observed in ICU length of stay and duration of mechanical ventilation. As a prolonged ICU admission is very well known to be associated with decreased quality of life,^5^ and also associated with the exposure, this causes residual confounding and therefore risk of bias of the results.

Additionally, quality of life after discharge from the ICU is a dynamic process and research shows that the outcomes studied tend to improve in patients over time.^6^ A later time-point (i.e. 12 months post discharge) might have been more appropriate as patients have progressed in their rehabilitation process, providing more final estimates of the outcomes after recovering from the ICU stay.

We share the authors’ enthusiasm for long-term follow-up of ECLS patients, yet believe this study was severely hampered by low enrolment and poor response rates. Studies seeking greater patient recruitment and higher response rates are therefore warranted to further our understanding of long-term ECLS outcomes.

## References

[1] de Vlugt R Spek B van de Pol I , et al. Quality of life after extra corporeal life support therapy. Perfusion 2022: 2676591221106148. 10.1177/02676591221106148 35656759

[2] Khan IR Saulle M Oldham MA , et al. Cognitive, psychiatric, and quality of life outcomes in adult survivors of extracorporeal membrane oxygenation therapy: A scoping review of the literature. Crit Care Med 2020; 48(10): E959–E970. 32886470 10.1097/CCM.0000000000004488

[3] Cheung KL Ten Klooster PM Smit C , et al. The impact of non-response bias due to sampling in public health studies: A comparison of voluntary versus mandatory recruitment in a Dutch national survey on adolescent health. BMC Public Health 2017; 17(1): 276. 28330465 10.1186/s12889-017-4189-8 PMC5363011

[4] Reinholdsson J Kraus-Schmitz J Forssblad M , et al. A non-response analysis of 2-year data in the Swedish Knee Ligament Register. Knee Surg Sports Traumatol Arthrosc 2017; 25(8): 2481–2487. 26724828 10.1007/s00167-015-3969-x

[5] Granja C Teixeira-Pinto A Costa-Pereira A . Quality of life after intensive care--evaluation with EQ-5D questionnaire. Intensive Care Med 2002; 28(7): 898–907. 12122528 10.1007/s00134-002-1345-z

[6] Busico M Intile D Sívori M , et al. Risk factors for worsened quality of life in patients on mechanical ventilation. A prospective multicenter study. Med Intensiva 2016; 40(7): 422–430. 26976118 10.1016/j.medin.2016.01.002

